# Influence of High Intensity Focused Ultrasound on the Microstructure and c-di-GMP Signaling of *Pseudomonas aeruginosa* Biofilms

**DOI:** 10.3389/fmicb.2020.599407

**Published:** 2020-12-15

**Authors:** Lakshmi Deepika Bharatula, Enrico Marsili, Scott A. Rice, James J. Kwan

**Affiliations:** ^1^School of Chemical and Biomedical Engineering, Nanyang Technological University, Singapore, Singapore; ^2^Singapore Centre for Environmental Life Sciences Engineering, Nanyang Technological University, Singapore, Singapore; ^3^Department of Chemical and Materials Engineering, Nazarbayev University, Nur-Sultan, Kazakhstan; ^4^School of Biological Sciences, Nanyang Technological University, Singapore, Singapore; ^5^Department of Engineering Science, University of Oxford, Oxford, United Kingdom

**Keywords:** *Pseudomonas aeruginosa*, biofilm, HIFU, microstructural effect, cyclic-di-GMP

## Abstract

Bacterial biofilms are typically more tolerant to antimicrobials compared to bacteria in the planktonic phase and therefore require alternative treatment approaches. Mechanical biofilm disruption from ultrasound may be such an alternative by circumventing rapid biofilm adaptation to antimicrobial agents. Although ultrasound facilitates biofilm dispersal and may enhance the effectiveness of antimicrobial agents, the resulting biological response of bacteria within the biofilms remains poorly understood. To address this question, we investigated the microstructural effects of *Pseudomonas aeruginosa* biofilms exposed to high intensity focused ultrasound (HIFU) at different acoustic pressures and the subsequent biological response. Confocal microscopy images indicated a clear microstructural response at peak negative pressures equal to or greater than 3.5 MPa. In this pressure amplitude range, HIFU partially reduced the biomass of cells and eroded exopolysaccharides from the biofilm. These pressures also elicited a biological response; we observed an increase in a biomarker for biofilm development (cyclic-di-GMP) proportional to ultrasound induced biofilm removal. Cyclic-di-GMP overproducing mutant strains were also more resilient to disruption from HIFU at these pressures. The biological response was further evidenced by an increase in the relative abundance of cyclic-di-GMP overproducing variants present in the biofilm after exposure to HIFU. Our results, therefore, suggest that both physical and biological effects of ultrasound on bacterial biofilms must be considered in future studies.

## Introduction

Bacterial biofilms are microstructured bacterial consortia. These bacterial cells display a high degree of physiological and topographical heterogeneity and grow on abiotic surfaces (e.g., biomedical devices) or biological surfaces (e.g., lung tissue) (Flemming et al., 2016). Bacteria within biofilms are more tolerant to antimicrobials compared to planktonic bacterial cells due to self-produced biofilm matrix consisting of extracellular polymeric substance (EPS), high bacterial concentration, exchange of genetic information in biofilms, differences in growth states of bacteria across the biofilm, and the expression of genes associated with tolerance or resistance to antimicrobial agents (Stewart and Costerton, 2001; Flemming and Wingender, 2010; Høiby et al., 2010). Given the increased tolerance of biofilms to antimicrobials, there remains a growing need for more effective antibiotics and new approaches to target biofilm infections. Developing novel antibiotics, however, is costly and time consuming. Thus, there have been increased efforts toward alternative approaches to induce cell death and/or promote dispersion of biofilms (Koo et al., 2017; Pinto et al., 2020).

One particularly promising approach for biofilm disruption is the use of therapeutic ultrasound, a non-invasive and cost-effective technique that provides targeted and localized mechanical effects (Erriu et al., 2014). Past research on the effect of ultrasound on biofilms has widely focused on low intensity ultrasound combined with antibiotics and/or cavitation agents (e.g., microbubbles). These studies showed that ultrasound-enhanced antibiotic treatment improved antibiotic efficacy, increased cell death, and reduced biofilm thickness (Lattwein et al., 2020). Interestingly, the application of ultrasound with microbubbles alone under these exposure conditions had minimal to no effect on biofilm removal (LuTheryn et al., 2019; Lattwein et al., 2020). In contrast, high intensity focused ultrasound (HIFU) without the addition of cavitation agents has also induced bacterial detachment from the substratum and left behind a patchy biofilm compared to the untreated control (Bigelow et al., 2009; Xu et al., 2012; Iqbal et al., 2013). Though HIFU alone was capable of disrupting biofilms, the biological effect on bacterial signaling molecules and/or cellular activity triggered by ultrasound exposure has not yet been investigated.

Here, we report on the physical and biological effects of HIFU on biofilms formed by *Pseudomonas aeruginosa*. The microstructural effects of HIFU were investigated through live/dead analysis, exopolysaccharide-lectin binding analysis, crystal violet assay and electrochemical response. Further, we investigated the impact of HIFU on the secondary messenger signaling system, cyclic diguanylate (c-di-GMP), which is a central regulator of the transition from the planktonic state to the biofilm and vice versa. In our study, we used a fluorescent bio-reporter strain of *P. aeruginosa* to quantify the effects of HIFU on biofilm formation and c-di-GMP signaling (Rybtke et al., 2012; Nair et al., 2017).

## Materials and Methods

### Biofilm Formation

A non-mucoid, green fluorescent protein marked strain of *P. aeruginosa* PAO1 (PAO1 Gfp) and a double labeled mutant strain of *P. aeruginosa* PAO1 that uses Gfp as indicator of the intracellular concentration of c-di-GMP and cyan fluorescent protein (Cfp) as a biomass indicator [PAO1 Tn7-Gm-eCFP P*_*cdrA*_: Gfp* (ASV)] were used for the experiments (Lee et al., 2014; Nair et al., 2017). The mutant strains and their applications have been summarized in Table 1. The bacterial cultures and biofilms were grown as previously described (Bharatula et al., 2020). Briefly, two sheets of 15 × 15 mm ITO:PET [UV-sterilized and cleaned with 70% ethanol (v/v)] were glued onto a sterile petri dish with silicone sealant (Selleys, Singapore). After curing, the petri dish was filled with 15 mL minimal medium with 6.9 mM glucose (ABTG) and inoculated with bacteria. The ABTG minimal medium (excluding casamino acids) was prepared according to the previous report (Chua et al., 2015a). To prepare the bacterial inoculum, *P. aeruginosa* was grown overnight in 10 mL of Luria Bertani Lennox (LB) broth for 16 h at 37°C and 200 rpm shaking (Zhu et al., 2019). Furthermore, the culture was centrifuged for 5 min and 4,629×*g* and re-suspended in 10 mL fresh ABTG medium. After inoculating at an optical density equivalent to 0.02 at 600 nm (UV-1280, Shimadzu UV-vis spectrophotometer), the petri dishes with ITO:PET sheets were incubated at 37°C and 50 rpm shaking for 72 h. The medium was replaced every 24 h with fresh medium.

**TABLE 1:** *Pseudomonas aeruginosa* strains used in this report.

**Strain**	**Application**	**References**
PAO1 *Gfp*	Analysis of biofilm viability using confocal microscopy (live/dead), crystal violet assay and electrochemistry for studies investigating the role of HIFU exposure at various acoustic pressures	Holloway et al., 1979
PAO1 P*cdrA*:*Gfp* (ASV)	Mutant where *cdrA* gene is fused with unstable Gfp plasmid; (i) Used for c-di-GMP quantification in biofilms after HIFU exposure (ii) Used for biomass determination in EPS staining study	Nair et al., 2017
PAO1 Δ*wspF* P*cdrA*:*Gfp* (ASV)	Mutant overproducing c-di-GMP; Used to study the role of c-di-GMP in biofilm after HIFU-treatment	Nair et al., 2017

### Acoustic Characterization of Biofilms

A leak proof, custom-made sample chamber was used to hold the biofilm coated ITO:PET sheets. The sample chamber with biofilm layer at the bottom was filled with ABTG medium and sealed with a Mylar sheet (Supplementary Figure 1). For HIFU treatment, 0.5 MHz transducer (H107, Sonic Concepts, Bothell, WA, United States) attached to a coupling cone was used in our experiments.

A needle hydrophone (0.2 mm diameter, Precision Acoustics, Dorset, United Kingdom) coupled with a submersible pre-amplifier and DC coupler was used to calibrate the measured pressure amplitude whereas, 1 mm diameter hydrophone (Onda Corporation, Sunnyvale, CA, United States) was used to study acoustic wave propagation for non-linearity determination. The hydrophone was adjusted, such that it coincided with the geometric focus of 0.5 MHz transducer (63.2 mm). For pressure calibration, the signal was triggered at varying input voltage ranging from 10 to 100 mV_pp_ for 20 cycles with a burst period of 10 ms. The resulting output voltage at each point was used to calculate the peak negative pressure using the following equation:

$$\mathrm{Pressure}\,\left(\mathrm{MPa}\right)=\frac{\mathrm{Output}\,\mathrm{Voltage}}{2\times \mathrm{Hydrophone}\,\mathrm{Sensitivity}}$$

Here, hydrophone sensitivity as defined by Precision Acoustics was 55.6 mV/MPa. For, non-linearity studies, the acoustic wave at peak negative pressure ranging from 0.5 to 5.5 MPa (as calculated from hydrophone calibration) was recorded.

For the biofilm exposure experiments, acoustic setup similar to previous study was adapted (Su et al., 2019). In short, sinusoidal wave electrical signal was triggered by function generator (Keysight 33210A) and amplified by high power RF amplifier (Electronics and Innovation, Rochester, NY, United States). The output signal was then converted to acoustic waves and transmitted to the target area by the HIFU transducer. An impedance matching network (Sonic Concepts, Bothell, WA, United States) was used to match the impedance of the HIFU to the output signal from the RF amplifier. The output emissions were received by a 7.5 MHz passive cavitation detector (PCD) (V320, Olympus, Singapore) aligned axially and laterally with HIFU transducer. The data was then collected by an oscilloscope (DSOX3032A, Keysight Technologies, Netherlands) or data acquisition board (DAQ) (PCI-5122, National Instruments, Texas, United States) (Supplementary Figure 1).

The biofilms were exposed for 3 min at 10% duty cycle and 50,000 cycles. The peak negative pressure amplitude was varied from 0.5 to 5.5 MPa (as calculated from hydrophone calibration). A sham sample was used as a control for all the HIFU exposed samples. The post processing of acoustic emissions to obtain power spectral density curve has been previously described (Jonnalagadda et al., 2020). Briefly, the post-processing involved extraction of power content from the fast Fourier transform of the received voltage signal. The power spectral density (PSD) differentiated between the presence of harmonics and broadband signal (Paliwal and Mitragotri, 2006). The presence of harmonics, sub-harmonics, and hyper-harmonics are typical for non-inertial cavitation whereas, broadband noise is usually indicative of inertial cavitation.

### Confocal Imaging of Biofilms

Following HIFU exposure, independent replicates were imaged to quantify the live/dead cells ratio, exopolysaccharide content and c-di-GMP response. The biofilm samples, irrespective of stains used were imaged as z-stacks under confocal laser scanning microscope (CLSM) (Zeiss LSM 780 inverted microscope; 20× resolution) at five separate locations within the region of acoustic focus. The 3 dimensional (3D) projections of the z-stacks were reconstructed using Imaris (Bitplane, Oxford Instruments).

### Live/Dead Cell Staining

To monitor bacterial cell viability, the biofilm samples (PAO1 *Gfp*) were stained with 8 μL of the *Baclight* live/dead stain and imaged by CLSM. The stock solution was made by adding 3 μL of both SYTO 9 (Excitation/Emission: 485/498) and propidium iodide (Excitation/Emission: 585/617) to 1 mL DI water.

### *cdrA* Correlated C-di-GMP Response

PAO1 P*_*cdrA*_*:*Gfp* and Δ*wspF* P*_*cdrA*_*:*Gfp* mutants were used to quantify the relative amounts of c-di-GMP in HIFU treated and control biofilms (Nair et al., 2017). Following HIFU exposure, the amount of cell biomass [cyan fluorescent protein (Cfp), Excitation/Emission: 435/485] and *cdrA* response [green fluorescent protein (Gfp), Excitation/Emission: 488/510], which is directly correlated to the amount of c-di-GMP, were imaged by CLSM. The Gfp/Cfp ratio, i.e., the *cdrA* correlated c-di-GMP response signal per biomass was quantified.

### Exopolysaccharide Staining

The PAO1 P*_*cdrA*__:_Gfp* mutant was used for these studies. The Cfp label of the mutant was used to image the biofilm cells. One mg/mL fluorescein isothiocyanate (FITC) conjugated concanavalin-A (con-A) (from *Canavalia ensiformis*, Sigma Aldrich, Singapore) in deionized water and calcofluor white stain (CWR) (Sigma Aldrich, Singapore), mixed with 10% KOH solution in a 1:1 ratio were prepared as stock solutions. Four microliter of con-A (Excitation/Emission: 492/518) and CWR (Excitation/Emission: 365/435) each were added to the biofilms to stain α polysaccharides and β polysaccharides, respectively. The biofilms were set aside for 30 min after addition of each stain.

### Image Processing and Quantification

All calculations were performed using MATLAB (Mathworks Inc.). Prior to any calculation, all the images were split into separate channels. Image processing steps involved Otsu’s image thresholding followed by image filtering using 2D median filtering (filter size = 5) (Yang et al., 2001; Beyenal et al., 2004b). The volumetric parameters were calculated as described by Beyenal et al. (2004a). We looked at parameters such as biovolume, porosity, and run-length. In summary, the biovolume was calculated from the following equation:

$$\mathrm{Biovolume}\,\left(\mu \,m^{3}\right)=\sum_{x=1}^{N_{x}}\sum_{y=1}^{N_{y}}\sum_{z=1}^{N_{z}}I\,m\,a\,g\,e\,S\,t\,a\,c\,k\,\left(x,y,z\right)\,v\,o\,x\,e\,l\,s\,i\,z\,e$$

The porosity of the biofilms was calculated as follows:

$$\mathrm{Porosity}=\frac{\mathrm{Total}\,\mathrm{no}.\mathrm{of}\,\mathrm{void}\,\mathrm{pixels}}{\mathrm{Total}\,\mathrm{no}.\mathrm{of}\,\mathrm{pixels}}$$

Run-length in a given direction is defined as the number of continuous biomass pixels. From these values, the aspect ratio was calculated in each axis as:

$$\mathrm{Aspect}\,\mathrm{Ratio}=\frac{X\,\mathrm{Run}-\mathrm{length}}{Z\,\mathrm{Run}-\mathrm{length}}$$

The quantification of Gfp/Cfp ratio (c-di-GMP per biovolume) and corresponding ratio images were obtained as suggested by previous report (Nair et al., 2017). To calculate the amount of c-di-GMP per cell biomass, the z-stacks of Cfp and Gfp channels corresponding to biomass and c-di-GMP were analyzed separately. A mask was created by thresholding and filtering the Cfp channel. This mask was multiplied with both Gfp channel and Cfp (graylevel) channel and a sum of the matrices was obtained. The ratio was calculated by dividing the Gfp value with Cfp value. This ratio was considered as the c-di-GMP per cells. The resulting heatmap from this ratio was termed as “ratiometric image.” The final ratio was further normalized with respect to the untreated control. For MATLAB code snippets see Supplementary Information.

### Crystal Violet Assay

The biofilm samples after HIFU test were washed with phosphate buffer saline (PBS) solution and further stained with 0.1% (v/v) crystal violet solution for 15 min. This was followed by washing the sheets with PBS, addition of 2 mL absolute ethanol and the measurement of absorbance at 550 nm using a microplate reader (TECAN M200, Switzerland).

### Electrochemical Monitoring

The setup previously described in Bharatula et al. (2020) was used. Briefly, a VSP or VMP3 multi-channel potentiostat (Bio-Logic, France) was connected to a three-electrode setup. The 15 × 15 mm ITO:PET sheet was used as working electrode and connected to a Pt sheet electrode holder as current collector (Latech, Singapore). The auxiliary and reference electrodes were a coiled titanium wire (Sigma Aldrich, Singapore) and Ag/AgCl standard electrode (Latech, Singapore), respectively. The three electrodes immersed in 15 mL fresh ABTG medium with 5 mM potassium ferricyanide as an exogenous redox mediator were connected to the potentiostat controlled by EC-Lab software (Bio-Logic, France). Electrochemical Impedance Spectroscopy (EIS) was carried out at open circuit potential (OCP), in the frequency range from 100 kHz to 30 mHz with sinusoidal potential of 10 mV amplitude. Furthermore, bias potential in the range to 50–500 mV vs. Ag/AgCl was applied to gain additional information on the biofilm electrochemical signature. The impedance data were fitted to an equivalent circuit model consisting of two resistor—constant phase element (CPE) blocks and an additional resistor in series using the Z-Fit feature in the EC-lab software (Bharatula et al., 2020).

### Colony Morphology Analysis

An area, representative of the acoustic focus in the samples exposed to HIFU and the untreated control sample was scraped and mixed in LB medium. Further, the solution was serially diluted till 10^5^ dilution was reached. Final volume of 100 μL of each dilution was individually plated on LB agar plates and incubated at 30°C for 24 h. After incubation, the colony forming units (CFU) were quantified using the formula:

$$\mathrm{CFU}=\frac{\mathrm{No}.\mathrm{of}\,\mathrm{counted}\,\mathrm{colonies}}{\mathrm{Dilution}\,\mathrm{factor}\times \mathrm{droplet}\,\mathrm{volume}}$$

Here, circular and wrinkled morphologies were counted separately. The percentage of CFU with wrinkled morphology was quantified as follows:

$$\%\mathrm{of}\mathrm{wrinkled}\mathrm{colonies}=\frac{\mathrm{CFU}\,\mathrm{of}\,\mathrm{wrinkled}\,\mathrm{morphology}}{\mathrm{Total}\,\mathrm{CFU}}\times 100$$

Furthermore, both the morphologies were imaged by light microscope (Carl Zeiss Primo star) at 4× magnification.

## Results

### Effect of HIFU on Biomass

**FIGURE 1:**
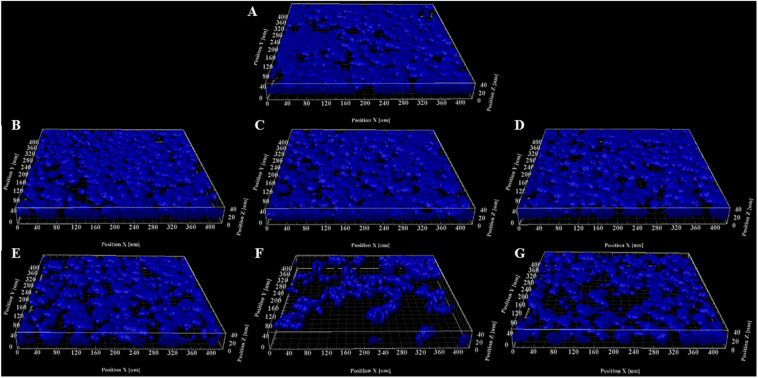
Representative 3D reconstructions of biofilms formed by PAO1 P*_*cdrA*_:Gfp* exposed to HIFU at **(A)** 0 MPa (Sham). **(B)** 0.5 MPa. **(C)** 1.5 MPa. **(D)** 2.5 MPa. **(E)** 3.5 MPa. **(F)** 4.5 MPa. **(G)** 5.5 MPa. The CFP channel of the confocal images of biofilms grown for 3 days and exposed to HIFU were converted to 3D reconstructions to observe the change in biomass.

**FIGURE 2:**
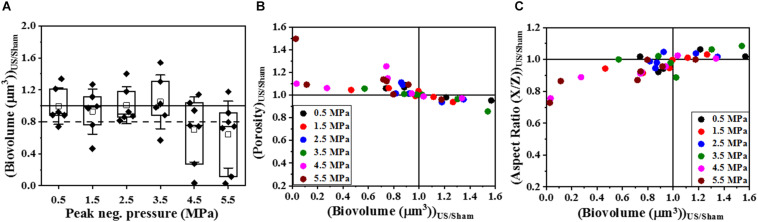
The quantitative analysis of volumetric changes in biofilms formed by PAO1 P*_*cdrA*_:Gfp* exposed to HIFU. **(A)** Change in biovolume with respect to acoustic pressure amplitude. An arbitrary threshold of 20% loss or more was considered significant. **(B)** Effect on biofilm porosity as the biovolume changed. **(C)** Effect on biofilm aspect ratio in X/Z direction as the biovolume changed.

To determine if HIFU induced a microstructural response in the biofilm, we quantified the biofilms formed by PAO1 mutant labeled with Cfp. Figures 1, 2 describe the qualitative and quantitative analysis of biovolume for untreated control and biofilms exposed to 0.5–5.5 MPa, respectively. Due to the heterogeneous nature of the biofilms and associated high variation in biomass values between replicates, a biovolume loss of 20% or more was arbitrarily considered as an estimate for the lower limit threshold for biofilm loss due to HIFU. Accordingly, a significant loss in biovolume, especially at high pressure amplitudes (4.5 and 5.5 MPa), was observed (Figures 1F–G, 2A). Further probing into volumetric properties revealed increase in porosity (Figure 2B) and decrease in aspect ratio in X vs. Z direction (Figure 2C) with respect to loss of biovolume on the substratum.

### Effect of HIFU on Biofilm Viability and EPS Components

Biofilms are comprised of cells as well as the extracellular matrix that holds them together and thus, biofilm control strategies may affect one or both components of the biofilm. As suggested by previous results, HIFU was able to disrupt the biomass yet its influence on the viability and the biofilm matrix remains unclear. Therefore, the effects of three pressure amplitudes on both the components were studied using confocal imaging, crystal violet assay, and electrochemical monitoring.

**FIGURE 3:**
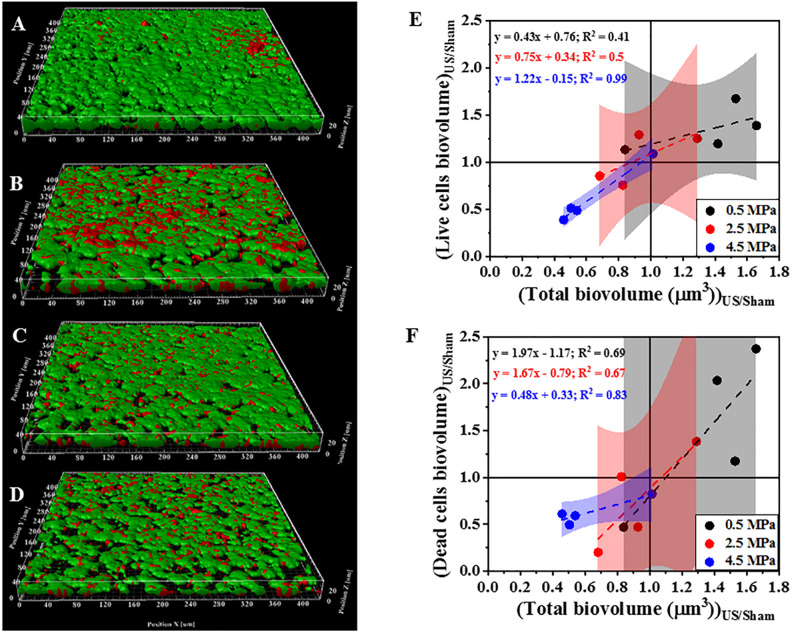
Representative 3D reconstructions from confocal images of biofilms formed by PAO1 *Gfp* exposed to HIFU stained at **(A)** 0 MPa (Sham). **(B)** 0.5 MPa. **(C)** 2.5 MPa. **(D)** 4.5 MPa. Green = live cells, Red = Dead cells. **(E)** Effect on biovolume of live cells with respect to change in total biovolume. **(F)** Effect on biovolume of dead cells with respect to change in total biovolume. The method was adapted to study role of HIFU on biofilm viability by staining with SYTO9 and PI. Here, SYTO9 stained live cells whereas, PI stained the dead cells. The quantification established a linear relationship between Live/dead cells biovolume and total cells biovolume. The linear equation and *R*^2^-value for individual regressions are indicated in the plots.

Figure 3 shows 3D reconstructions of confocal images of biofilms exposed to HIFU and the subsequent effect of HIFU on cell viability marked by live/dead staining. Based on 3D reconstructions (Figures 3A–D) and image quantification (Figure 3E) of the untreated biofilm and HIFU treated biofilms, there was no significant effect of HIFU at 0.5 and 2.5 MPa on live biofilm biomass. When the pressure was increased to 4.5 MPa, there was a reduction in live biomass. Interestingly, the dead biomass was also reduced alongside the live biomass especially after exposure at 4.5 MPa (Figure 3F).

**FIGURE 4:**
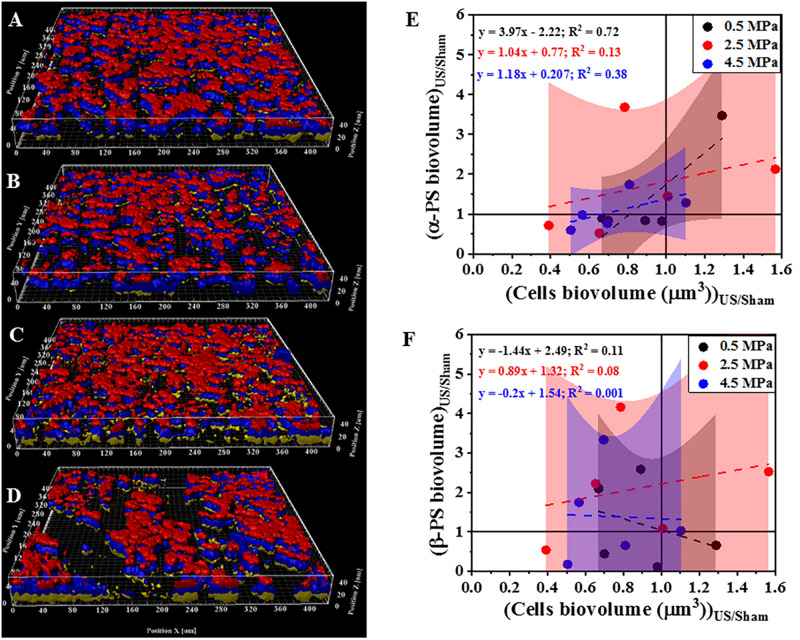
Representative 3D reconstructions of confocal images of biofilms formed by PAO1 P*_*cdrA*_:Gfp* and exposed to HIFU at **(A)** 0 MPa (Sham). **(B)** 0.5 MPa. **(C)** 2.5 MPa **(D)** 4.5 MPa. Blue = Biofilm cells, Red = Con-A, Yellow = CWR. **(E)** Effect on biovolume of α-polysaccharides with respect to bacterial cells biovolume. **(F)** Effect on biovolume of β-polysaccharides with respect to cell biovolume. PS in plots refers to polysaccharides. The method was adapted to study role of HIFU on biofilm exopolysaccharides by staining with Con-A and CWR. The CFP channel of the PAO1 P*_*cdrA*_:Gfp* biofilms indicated the change in biomass whereas, Con-A stained α-polysaccharides and CWR stained the β-polysaccharides. The quantification established a linear relationship between α-/β-polysaccharides biovolume and total cells biovolume. The linear equation and *R*^2^-value for individual regressions are indicated in the plots.

Next, we investigated the effect of HIFU treatment on the distribution and quantity of polysaccharides that comprise part of the *P. aeruginosa* biofilm matrix (Figure 4). Two fluorescent dyes were used to distinguish between two broad classes of polysaccharides. Specifically, Con-A was used to visualize and quantify the α-polysaccharides and CWR was used to identify the β-polysaccharides. Based on 3D reconstructions of the biofilm (Figures 4A–D) it was observed that the bacterial cells (blue) were always surrounded by the α-polysaccharides (red) at the bottom and β-polysaccharides (yellow) on top irrespective of the HIFU pressure applied. Quantitative image analysis (Figure 4E) indicated a positive linear relationship between relative α-polysaccharides biovolume and the remaining biofilm cells. This observation suggested that α-polysaccharides were removed with the bacterial cells after HIFU exposure. In contrast, the relative β-polysaccharides volume was less reduced, suggesting that β-polysaccharides may remain in the biofilm micro-environment irrespective of cell biomass loss (Figure 4F).

**FIGURE 5:**
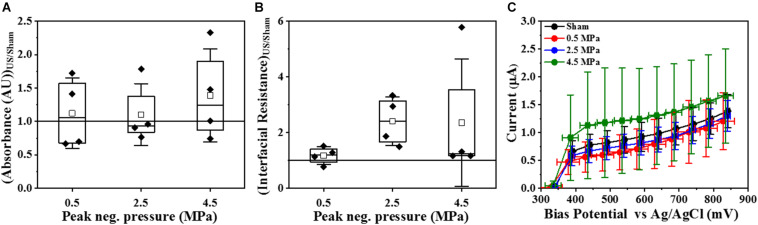
Effect of HIFU on the entire biofilm surface. **(A)** Crystal violet absorbance at 550 nm for biofilms formed by PAO1 *Gfp* exposed to HIFU at various acoustic pressures. **(B)** Interfacial Resistance obtained by EIS and fit to an equivalent circuit and **(C)** Faradaic current characteristics at increasing bias potential for biofilms formed by PAO1 *Gfp* exposed to HIFU at various acoustic pressures. The results are statistically insignificant.

Crystal violet assay and electrochemical monitoring (Figure 5) were used to corroborate the effect of HIFU exposure on the viability and biofilm matrix. In contrast to confocal imaging which analyzed smaller areas, these techniques allowed us to investigate the entire surface area exposed to HIFU. The crystal violet assay (Figure 5A) indicated no apparent effect of HIFU at any given pressure on the crystal violet absorbance. EIS allows for the rapid measurement of microstructural changes in the biofilm after ultrasound exposure through the variation of parameters such as interfacial resistance under open circuit potential conditions and current output at controlled electrode potential. Here, a marked yet, statistically insignificant increase in the interfacial resistance with respect to the sham (Figure 5B) was observed at all pressures. Even so, there was no significant difference between the current for sham and HIFU treated biofilms (Figure 5C), there was a slight (albeit not statistically significant) increase in current for 4.5 MPa exposed biofilms.

### Effect of HIFU on *cdrA* Correlated c-di-GMP per Biovolume Ratio

**FIGURE 6:**
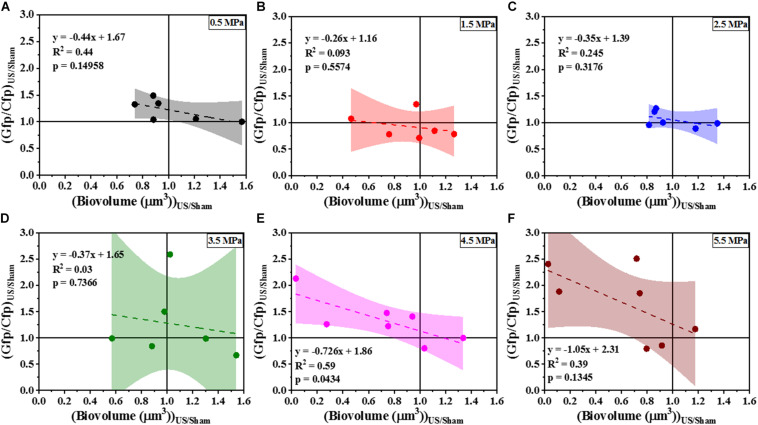
The correlation of Gfp/Cfp ratio and biofilm biovolume at various pressure amplitudes normalized to the sham—**(A)** 0.5 MPa. **(B)** 1.5 MPa. **(C)** 2.5 MPa. **(D)** 3.5 MPa. **(E)** 4.5 MPa. **(F)** 5.5 MPa. The quantification established a linear relationship between Gfp/Cfp ratio (indicative of *cdrA* correlated c-di-GMP per unit cell biovolume) and cells biovolume. The linear equation, *R*^2^-value and *p*-value for individual regressions are indicated in the plots. Here *p*-value is the significance level at which the obtained slope is statistically different from zero.

To determine whether the loss in biovolume was a result of a purely physical effect or due to changes in gene regulation that controls biofilm formation, the response of the Gfp channel corresponding to the Cfp channel (seen in Figure 1) was analyzed using an indirect c-di-GMP quantification technique. Here, the *cdrA-*Gfp signal is a proxy for the intracellular c-di-GMP concentration. Quantitative image analysis (Figure 6) of the confocal images revealed the dependence of change in ratiometric signal on the loss of biofilm biomass. Figures 6A–F shows the scatter plot of Gfp/Cfp ratio with respect to remaining cell biovolume at six different acoustic pressure amplitudes. The increase in the ratio was evident in most samples (∼ 4 out of 7 samples) exposed at 4.5 and 5.5 MPa especially when the biovolume loss was above the 20% biovolume loss threshold. Statistical significance was not the best way to characterize the changes in these experiments as both cavitation and biofilm growth are subject to have levels of variability. To better understand the influence of acoustic pressure amplitude on the relationship between biovolume and Gfp/Cfp ratio, the data points were fit linearly at individual pressures. A negative correlation between biovolume and ratio was significant at 4.5 and 5.5 MPa whereas biofilms exposed to HIFU at pressure amplitudes below 4.5 MPa did not show any substantial change in Gfp signal per cell of the remaining cells in the biofilm. At *p* = 0.05, only the slope of 4.5 MPa response was statistically different from zero. All the *p*-values are shown in the respective plots along with the linear equation.

**FIGURE 7:**
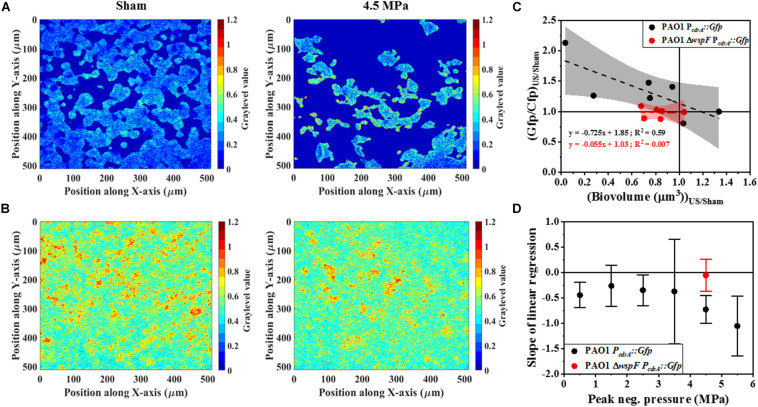
Representative ratiometric images (i.e., images of Gfp channel per Cfp channel) of biofilms formed by **(A)** PAO1 P*_*cdrA*_:Gfp* exposed to HIFU at 0 MPa (Sham) and 4.5 MPa. **(B)** Representative ratiometric images of biofilms formed by c-di-GMP overproducing PAO1 Δ*wspF* P*_*cdrA*_:Gfp* exposed to HIFU at 0 MPa (Sham) and 4.5 MPa. The colorbar shows the graylevel value, i.e., the Gfp/Cfp ratio in the spatial plane. **(C)** The correlation of Gfp/Cfp ratio and biofilm biovolume for biofilms formed by PAO1 P*_*cdrA*_:Gfp* and PAO1 Δ*wspF* P*_*cdrA*_:Gfp* exposed to HIFU at 4.5 MPa and normalized to the sham. The linear equation and *R*^2^-value for individual regressions are indicated in the plots. **(D)** Variation in the slope of linear regression with increasing acoustic pressure amplitude for biofilms formed by PAO1 P*_*cdrA*_:Gfp* and PAO1 Δ*wspF* P*_*cdrA*_:Gfp*.

Next, we compared the effect of HIFU induced biofilm removal at 4.5 MPa and subsequent effect on *cdrA* correlated c-di-GMP response in wild type-PAO1 P*_*cdrA*_:Gfp* and a Δ*wspF* mutant that overproduces c-di-GMP as a consequence of the mutation, i.e., PAO1 Δ*wspF* P*_*cdrA*_:Gfp* (Figure 7). It is to be noted that the Δ*wspF* biofilms were thicker than the ones formed by wild type strain (Supplementary Figure 3). Supplementary Figure 3 shows the comparison of biofilm growth by wild type and Δ*wspF* strains. Here, the Δ*wspF* biofilms both untreated (Supplementary Figure 3C) and HIFU-treated (Supplementary Figure 3D) were approximately four times thicker than the ones formed by wild type strain (Supplementary Figures 3A,B). The biovolume quantification (Supplementary Figure 3E) shows that HIFU exposure decreased the biovolume in both strains yet, contrary to wild type that removed cells from the base, in Δ*wspF* just layers of cells were removed from the top.

Figures 7A,B shows the qualitative analysis in the form of representative ratiometric images of Gfp per Cfp signal expressed by untreated biofilms and HIFU-treated biofilms for PAO1 P*_*cdrA*_:Gfp* and PAO1 Δ*wspF* P*_*cdrA*_:Gfp*, respectively. Inspection of images in Figure 7A indicated that the ratiometric signal in wild type biofilms gradually increased as HIFU acoustic pressures amplitude increased to 4.5 MPa. The ratiometric images of biofilms at other acoustic pressure amplitudes is shown in Supplementary Figure 2. In contrast, the qualitative image (Figure 7B) analysis of Δ*wspF* mutant biofilms at 4.5 MPa showed no increase in the ratiometric signal compared to wild type biofilms (Figure 7A). The same was reflected in the quantitative Gfp/Cfp ratio and biovolume relationship (Figure 7C). Here, the curve of PAO1 P*_*cdrA*_:Gfp* is the same as Figure 6E; it was added here for a better comparison.

Figure 7D shows the slope obtained from the linear regression for various acoustic pressure amplitudes. Here, the slope of linear regression was always less than zero indicating an increase in the Gfp/Cfp even at the lowest acoustic pressure. Nonetheless, a decreasing trend was observed as the pressure increased. Interestingly, the slope tracked back to zero for the biofilms formed by Δ*wspF* mutant.

**FIGURE 8:**
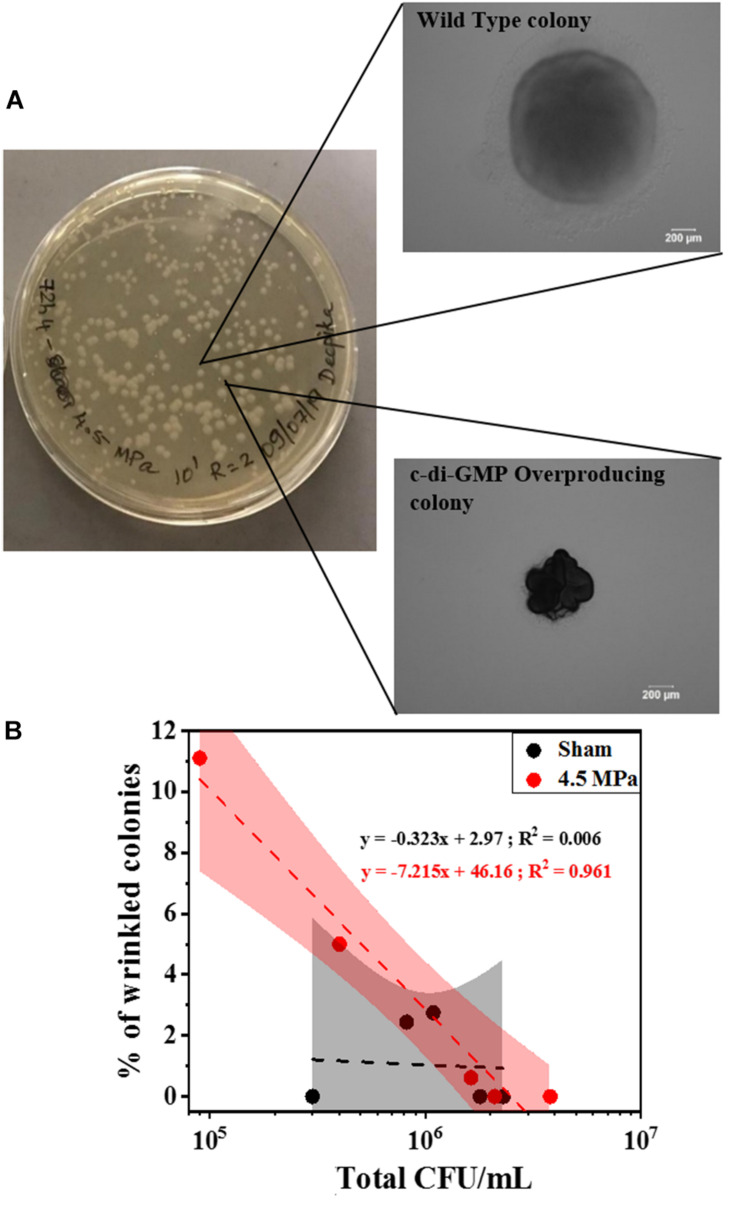
This analysis is related to emergence of wrinkled colonies (indicative of c-di-GMP overproduction) after HIFU exposure at 4.5 MPa. **(A)** Representative optical images of various colony variants found in the biofilms formed by PAO1 P*_*cdrA*_:Gfp* after exposure to HIFU at 4.5 MPa. **(B)** Linear relationship between total CFU and % of wrinkled colonies after sham and 4.5 MPa HIFU treatment. The linear equation and *R*^2^-value for individual regressions are indicated in the plots.

To corroborate the changes in c-di-GMP, we further investigated the genotypic composition of the biofilms by colony morphology analysis. Specifically, we looked for the presence of wrinkled colony morphology variants indicative of high c-di-GMP production due to mutations in the *wsp* pathway (Figure 8A). A positive correlation between the percentage of wrinkled colonies (Figure 8B) and total CFU was observed for 4.5 MPa treated biofilms whereas, the slope of untreated biofilms was not significantly different from zero. This suggests that at the higher acoustic pressure, where loss of the biofilm was observed, the remaining biofilm appears to have a higher relative abundance of wrinkled variants that overproduce c-di-GMP. This may explain the observations above (Figure 7) where we observe an increase in relative c-di-GMP production after exposure to increased acoustic pressures.

### Role of HIFU Properties in Microstructural and Biological Changes

Since our aim was to determine the influence of ultrasound alone on the biofilm, we used HIFU to achieve the desired effects. Introducing a coupling cone to the experimental system exhibited more intense power at the focus (Supplementary Figure 4A) compared to free-field (Supplementary Figure 4B). This pressure distribution calibration in presence of coupling cone also shows that the HIFU focus is limited to ∼3 mm in the radial plane.

Furthermore, the changes in acoustic properties at the pressures with maximum microstructural and biological response was apparent. The cavitation properties were investigated using PSD curve. For the frequency content, the raw data obtained from the oscilloscope was post processed and converted to a PSD curve. Here, inertial cavitation response was observed at 3.5 MPa and higher, in the representative PSD (Supplementary Figure 5). It is to be noted that this analysis just described inertial cavitation and there was a possibility of non-inertial cavitation which was revealed in the frequency content of the unfiltered signal. Investigations of unfiltered data revealed sub-harmonic signal at higher pressures indicating stable cavitation (Supplementary Figure 6).

Emergence of a non-linear acoustic wave was also investigated as a possible mechanism for the changes in biofilm. In contrast to the wave propagation in free-field (Supplementary Figure 7A), the distortion of rarefactional acoustic waves above 4.5 MPa indicating non-linear wave propagation was evident in a coupling cone setup (Supplementary Figure 7B).

## Discussion

Using therapeutic ultrasound on bacterial biofilms is gaining momentum as an efficient treatment strategy. To observe any effects, the choice of acoustic parameters is crucial (Brayman et al., 2017). While low intensity ultrasound in combination with microbubbles and antibiotics is intended to kill bacteria, HIFU treatment specifically breaks down and disrupts the biofilm, but does not necessarily rely on killing to achieve these effects (Erriu et al., 2014). At low acoustic intensity, exposing *P. aeruginosa* biofilms to varying ultrasonic frequency did not show any significant change in biofilm viability (Qian et al., 1997). In contrast, previous studies at high intensity have shown that varying parameters such as duty cycle, burst period and exposure time of HIFU cause loss of biomass in *P. aeruginosa* and *Enterococcus faecalis* biofilms (Xu et al., 2012; Iqbal et al., 2013). All of these studies revealed a vital role of acoustic intensity in the disruption of biofilms. Since our aim was to determine the influence of ultrasound alone on the biofilm we used HIFU to achieve the desired effects.

Furthermore, the mechanical effects of HIFU such as cavitation and acoustic streaming that might drive the disruption process rely strongly on the peak negative pressure (Mo et al., 2012). However, further investigation is required in the role of varying acoustic pressure amplitude on biofilms (Vyas et al., 2019). A study on acoustic pressure variation on *Escherichia coli* biofilms showed a reduction in CFU at relatively high pressures (Bigelow et al., 2009). Our studies showed biovolume loss at higher pressures and were consistent with Bigelow et al. (2009). Additional volumetric parameters such as porosity and aspect ratio gave a better understanding of the mechanical changes in biofilms after HIFU exposure (Lewandowski and Beyenal, 2007). The increase in porosity evidenced by the 3D reconstructions and quantification of biofilms indicated that HIFU penetrated the biofilm and uprooted the cells from the base of the substratum. Furthermore, the change in the aspect ratio of the run lengths confirmed that HIFU distorted the microcolonies in z-direction (Beyenal et al., 2004a).

Once we established that HIFU influenced the biofilm cells, we investigated the contribution of viable/non-viable cells and EPS to the microstructural change. Our findings showed that HIFU was able to detach both live and dead cells from the ITO:PET surface especially at 4.5 MPa. The biofilm detachment by HIFU was in agreement with previous studies (Bigelow et al., 2009; Xu et al., 2012; Iqbal et al., 2013). Additionally, there was no drastic increase in the dead cells after HIFU exposure; cell death was not a primary consequence of HIFU in contrast to the effects observed in presence of external agents such as microbubbles and antibiotics (Erriu et al., 2014; Cai et al., 2017; Lattwein et al., 2020). Our results therefore suggest that acoustic intensity was linked to the physical disruption of biofilms.

Next, we investigated the effect of ultrasound on exopolysaccharides. In addition to the bacterial cells, the biofilm matrix forms a major part of a biofilm (Flemming and Wingender, 2010). Previously, it has been suggested that low intensity ultrasound significantly affected polysaccharide synthesis expression in *Staphylococcus epidermidis* and *P. aeruginosa* biofilms (Zhu et al., 2013; Zhang et al., 2019). A positive correlation between EPS (stained by con-A) and biomass loss after low intensity ultrasound exposure in presence of microbubbles has also been determined (Agarwal et al., 2014). However, the impact of HIFU on the biofilm matrix has not yet been well studied (Pinto et al., 2020). In our study, con-A and CWR stains were used to study the role of HIFU on biofilm exopolysaccharides (Baird et al., 2012). These are general lectin-based polysaccharide stains and have been previously used to study exopolysaccharides in both biofilms (Baird et al., 2012) and aerobic granules (Chen et al., 2007).

In principle, each monosaccharide unit in the biofilm carbohydrates consist of α and β glycosidic linkages that bond with another monosaccharide or molecule (for e.g., lectins). Here, α and β linkages are stereoisomers where, the α-glycosidic bond is formed when the binding carbons have the same stereochemistry and β-glycosidic bond occurs when the two carbons have different stereochemistry. The biofilm exopolysaccharides are rich in such α and β linkages that bind to con-A and CWR, respectively (Baird et al., 2012).

In *P. aeruginosa*, biofilms formed by the non-mucoid PAO1 strain primarily consist of two exopolysaccharides namely, Pel, a glucose rich polysaccharide and Psl, a pentasaccharide repeat structure consisting of mannose, glucose and galactose (Mann and Wozniak, 2012). Here, con-A has a binding specificity toward α-mannose and α-glucose (Strathmann et al., 2002). Similarly, *P. aeruginosa* consists of cellulose-resembling β-1,4 and β-1,3 glucose units that binds with CWR (Stewart et al., 1995). To this effect, con-A and CWR stains are not specific to Pel or Psl, rather they give a combined response of both the exopolysaccharides by bonding with α and β-glycosidic link in the structure of the sugar.

Our findings revealed that HIFU was able to remove the bacterial cells and the α-polysaccharides linked to them, whereas removal of β-polysaccharides located at the top of the biofilm did not depend on the biovolume removal. This indicated two possible effects of HIFU on α-polysaccharides: (i) HIFU interacted with biofilm exopolysaccharides and degraded them or (ii) they were simply removed from the surface along with the bacterial cells due to their strong linking as a result of active or passive dispersal; both resulting in destabilizing the biofilm microstructure (Kaplan, 2010; Agarwal et al., 2014). Based on the aforementioned indication, con-A bonded with two major glycosyl units in *P. aeruginosa*, i.e., mannose (formed ∼20% of Psl structure) and glucose (dominant in Pel and formed ∼13% of Psl) (Ma et al., 2007; Jennings et al., 2015). As a result, the disruption of α-polysaccharides suggested the ability of HIFU to affect both Pel and Psl exopolysaccharides in *P. aeruginosa.*

Although we investigated the effect of HIFU on cells and EPS sugars, these changes alone do not hint at total biofilm removal because the microstructure of a biofilm is dynamic and complex (Flemming and Wingender, 2010). Therefore, to further understand the relationship between HIFU and total biofilm dispersal, we used a common method for quantifying acoustic-biofilm interactions, crystal violet assay (He et al., 2011; Zhu et al., 2013; Dong et al., 2017; Koibuchi et al., 2018). Crystal violet is known to stain the entire biofilm, i.e., live cells, dead cells, and extracellular matrix (Merritt et al., 2005). We also utilized EIS to characterize changes in the biofilm biomass and structure as it has been found to be an efficient tool to detect the growth and disruption of biofilms on conductive surfaces (Dominguez-Benetton et al., 2012). The EIS response of *P. aeruginosa* biofilms grown on ITO:PET substrate suggested that interfacial resistance and current characteristics indirectly measure the viability of the biofilm (Bharatula et al., 2020). The current characteristics are also influenced by Psl in the EPS. The interfacial resistance agreed with the live/dead stain results. Based on the non-removal of β-polysaccharides in confocal analysis, crystal violet assay, and current at bias potential, no significant change in biofilms with respect to acoustic pressure indicated that biofilm matrix components remained on the substratum.

Another possibility for the insignificant changes in the crystal violet assay and electrochemical monitoring was that the microstructural effects were a localized phenomenon. The beam focus of a 0.5 MHz HIFU transducer is 3 mm in radial diameter (as observed by the pressure distribution map), resulting in an exposure area of ∼7 mm^2^ (Bazan-Peregrino et al., 2012). Although this is a relatively small area compared to the entire area covered by biofilm (225 mm^2^), it is easily visualized using confocal microscopy. Crystal violet assay and EIS, however, analyze an area that extends beyond the focus of the HIFU beam Thus, these sampling methods provide insight on the effects of HIFU on regions within and beyond the focus of the HIFU, and thus suggested that the results we observed were restricted to only biofilm within the acoustic focus of HIFU.

From the microstructural results, HIFU removed large regions of the biofilm, however, the biological mechanism behind such observation remains undefined. A previous study has suggested that changes in genetic factors such as quorum sensing, protein metabolism, and motility are possible after exposure to low intensity ultrasound (Zhang et al., 2019). We focused on the impact of HIFU on a key regulatory system that controls biofilm formation and dispersal and is known to be linked to cellular responses to environmental cues such as changes in oxygen, nutrient concentrations, as well as nitric oxide that lead to dispersal (Valentini and Filloux, 2016). C-di-GMP is a secondary messenger that plays a key role in regulating the shift between planktonic and biofilm bound cells (Ha and O’Toole, 2015). Moreover, c-di-GMP is crucial in regulating the stress response in *P. aeruginosa* (Chua et al., 2015b). The intracellular c-di-GMP was quantified using a reporter strain that responds to changes in c-di-GMP concentrations by inducing Gfp production from the promoter of the *cdrA* gene (Nair et al., 2017). The fluorescent bio-reporter used in this study was specifically developed for the indirect characterization of the c-di-GMP from planktonic bacteria and biofilms using confocal microscopy (Rybtke et al., 2012; Nair et al., 2017). Previous optimization of the bio-reporter strain showed that the data gathered from the confocal microscope correlated well with the chemical quantification (Nair et al., 2017). This correlation confirmed that the bio-reporter used was an efficient indicator of the c-di-GMP levels within the cells in different parts of the biofilms and at different time. Moreover, the c-di-GMP concentration may vary throughout the biofilm due to physiological heterogeneity. Therefore, this characterization technique provided information about the spatial and temporal distribution of c-di-GMP.

In principle, the amount of c-di-GMP per biovolume is independent to the amount of biomass loss due to HIFU if the removal of biofilm is truly non-specific. At low acoustic pressure amplitudes before 3.5 MPa, this principle held true. However, our findings revealed an increase in *cdrA* dependent c-di-GMP response per remaining biomass at acoustic pressure amplitudes at and above 4.5 MPa, which corresponded to more consistent biofilm removal. It was therefore possible that the biofilm had c-di-GMP hot-spots that were detectable when “weaker” portions of the biomass was removed.

One possible mechanism for this change entirely relies on mechanical disruption of either the matrix or cells to cause biofilm disaggregation. Alternatively, the cells actively respond to the acoustic stresses from ultrasound to disperse suggesting ultrasound may induce changes in gene expression that result in active dispersal of the bacterial cells from the biofilm (Kaplan, 2010). The mechanism of the changes was tested by treating biofilms formed by Δ*wspF* mutation that overproduce c-di-GMP. Such biofilms often contain spontaneous mutants that do not disperse as effectively as the wild-type cells and are characterized by small colony variants with wrinkled morphology (Hickman et al., 2005).

The response from biofilms formed by Δ*wspF* mutation did not show any change in c-di-GMP despite the removal of bacterial cells. Here, the lack of dispersal suggested that the genetic signal cascade that regulated the dispersal was blocked. In comparison, a clear change was observed in biofilms formed by wild type strain indicating the influence of a biological mechanism as opposed to a purely mechanical one. Moreover, the increase in c-di-GMP per biomass ratio (Gfp/Cfp ratio) in the wild type biofilms after HIFU treatment suggested a slow transition toward PAO1 Δ*wspF* biofilm condition. Hence, an understanding of the response of high c-di-GMP producing biofilms toward HIFU was necessary. The lack of c-di-GMP response in PAO1 Δ*wspF* biofilms indicated that biofilms may develop resilience to HIFU as they become stronger.

Furthermore, the colony morphology studies corroborated the confocal microscopy findings where an increase in c-di-GMP was observed in wild type biofilms. Our findings revealed the c-di-GMP response was a consequence of an increase in genotypic variants (in the form of wrinkled colonies) with naturally high c-di-GMP production. Here, it is possible that HIFU induced dispersal of wild type cells, leaving behind the wrinkled variants that overproduced c-di-GMP. Several consequences of changes in c-di-GMP concentration in a biofilm have been previously reported. In *P. aeruginosa*, changes in c-di-GMP resulted in functional changes in LapG, which then cleaved *cdrA* from the surface of the cell. Since Pel is also attached to *cdrA*, this polysaccharide is then released from the cell surface to enable dispersal (Rybtke et al., 2015). Moreover, increased c-di-GMP levels were also shown to inversely affect the quorum sensing regulated *rhl* and *pqs* systems (Chua et al., 2017). Thus, it is plausible that biofilms have a biological response to the mechanical stress from HIFU.

Our investigations also revealed that the mechanical stress by HIFU at higher pressures was probably due to two independent mechanisms: (i) non-linearity of the acoustic wave and (ii) inertial and stable cavitation leading to fluid streaming effects and acoustic radiation forces that exert shear stress on the biofilms (Nyborg, 2006; Stride and Coussios, 2019). Previously, the application of external shear in the form of fluid flow has shown increase in the c-di-GMP levels in planktonic *P. aeruginosa* and was associated with increased biofilm development (Rodesney et al., 2017). Thus, it is possible that HIFU was sensed through similar mechanosensing mechanisms. However, studies on bacterial mechanosensing are limited to the planktonic cells and its effect on biofilm is unknown (Gordon and Wang, 2019). It has been suggested that either changes in rotation of the flagella or the membrane protein PilYI may act as mechanosensors in *P. aeruginosa* and further work is needed to determine if these bacterial components are also involved in HIFU sensing (Siryaporn et al., 2014; Luo et al., 2015).

Although the results are promising, there are few limitations to our study. As the characterization techniques used were destructive, a before and after effect of ultrasound on the same sample was not possible. Instead, an untreated biofilm grown from the same overnight culture was considered as a control. This control may not exactly resemble the treated sample resulting in variability in our observations. Additionally, there were limitations for the transcriptomic and/or nucleotide analysis of the HIFU-treated biofilms (which may give more insight into the biological response) due to the amount of biomass available in the test system. HIFU is used in biological applications to focus sound waves to create a small volume of intense acoustic energy, thereby avoiding off-target side effects. Here, the focus is not a point in space but a small elliptical volume resembling a grain of rice with maximum intensity at the center. To reiterate, for a 0.5 MHz transducer, the focus has a 3 mm radial diameter (Bazan-Peregrino et al., 2012). Therefore, we believe that the microstructural and biological changes were localized to this area as was evident from the electrochemical and crystal violet data. Therefore, we are constrained to the volume of biofilm exposed to HIFU to study its effects. This localization amounted to a miniscule volume of bacteria extracted and post-processed for biological characterization thereby, limiting a direct quantification of c-di-GMP levels in the system. As a result, an indirect although efficient fluorescent bio-reporter was used. Future studies (beyond the scope of this report) will explore: (i) other acoustic parameters (duty cycle, HIFU frequency), (ii) different HIFU transducers (for e.g., multi-element transducers with focus of 6 mm radial diameter), (iii) introduction of cavitation agents (for e.g., polymeric nano-cups or multi-cavity shells) (Kwan et al., 2015; Su et al., 2019), and (iv) switching to a relatively dynamic flow-cell system for biofilm growth (Sternberg and Tolker-Nielsen, 2006) to address the limitations.

## Conclusion

In summary, acoustic pressures equal to or greater than 4.5 MPa were optimal to observe HIFU-biofilm interactions. The cell viability studies showed that HIFU at 4.5 MPa removed bacteria from the surface although the complete removal was never achieved. Staining the exopolysaccharides revealed that HIFU penetrated the β-polysaccharides and was able to remove and/or degrade α-polysaccharides. Although, biofilm cells were removed from the surface, components of biofilm matrix still remained as observed from crystal violet and EIS studies. The most prominent observation was increase in the c-di-GMP signal after HIFU exposure suggesting that the remaining biofilms have a biological response to HIFU. Therefore, while investigating mechanical/physical approaches on biofilm, looking at the removal of biomass and change in microstructure is not sufficient. It is vital to track the transcriptomic response of the surviving biofilm in the future and our results are a first step toward showing the importance of such biological changes.

## Data Availability Statement

The raw data supporting the conclusions of this article will be made available by the authors, without undue reservation.

## Author Contributions

LDB, SR, and JK conceptualized the experiments for Figures 1–4, 6–8. LDB, EM, and JK conceptualized and analyzed the experiments in Figure 5. LDB performed the experiments, produced Figures 1–8 and wrote the manuscript. JK and LDB wrote the MATLAB codes for confocal image analysis. All authors reviewed and edited the manuscript.

## Conflict of Interest

The authors declare that the research was conducted in the absence of any commercial or financial relationships that could be construed as a potential conflict of interest.
