# Supplementary material for: Influence of High Intensity Focused Ultrasound on the Microstructure and c-di-GMP Signaling of Pseudomonas aeruginosa Biofilms
Source: Front Microbiol. 2020 Dec 15;11:599407. doi: 10.3389/fmicb.2020.599407 (PMC7769819; doi:10.3389/fmicb.2020.599407)
Supplement: Supplementary file 1 [file Data_Sheet_1.PDF]

Supplementary Information

1 Supplementary Figures

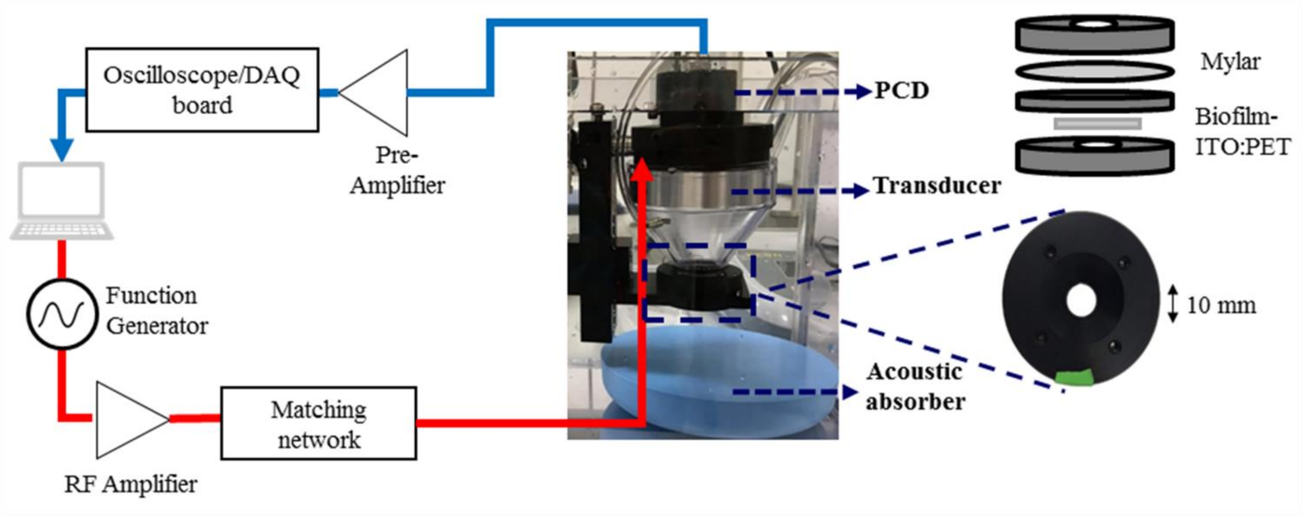

Supplementary Figure 1. Schematic of the HIFU setup and the custom-made sample chamber used in the experiments.

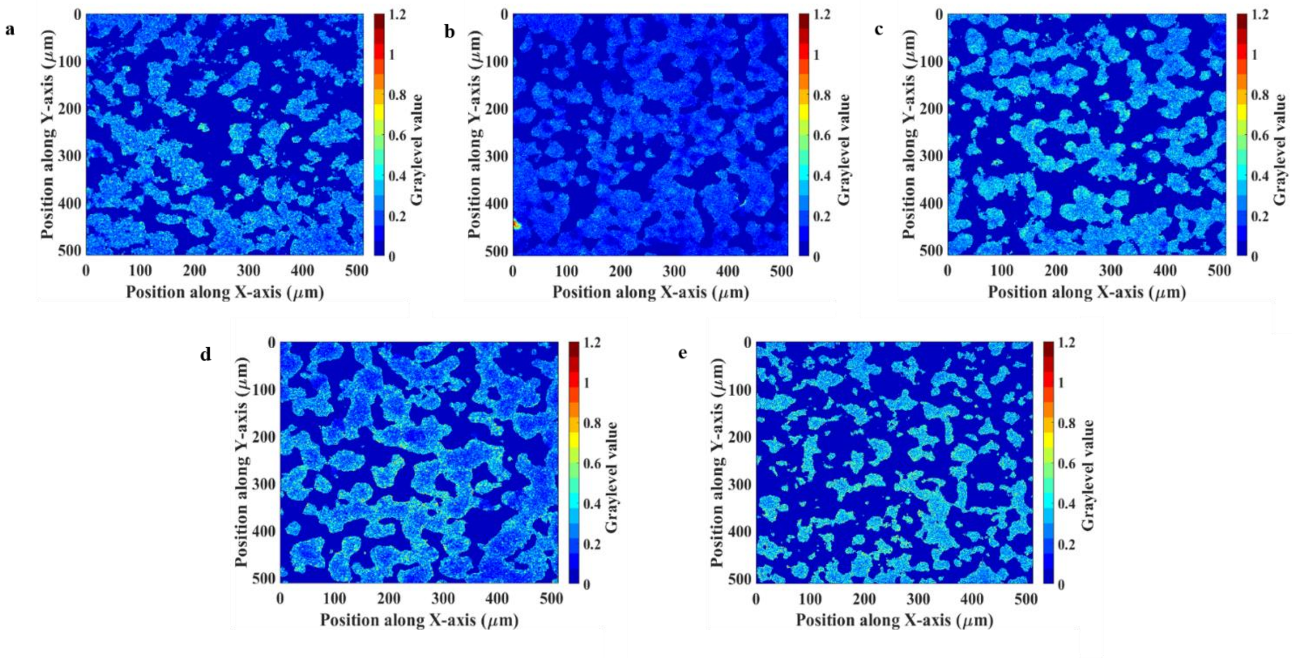

Supplementary Figure 2. Representative ratiometric (Gfp/Cfp) images of biofilms formed by PAO1 P_{cdrA}::Gfp exposed to HIFU at **a:** 0.5 MPa **b:** 1.5 MPa **c:** 2.5 MPa **d:** 3.5 MPa **e:** 5.5 MPa.

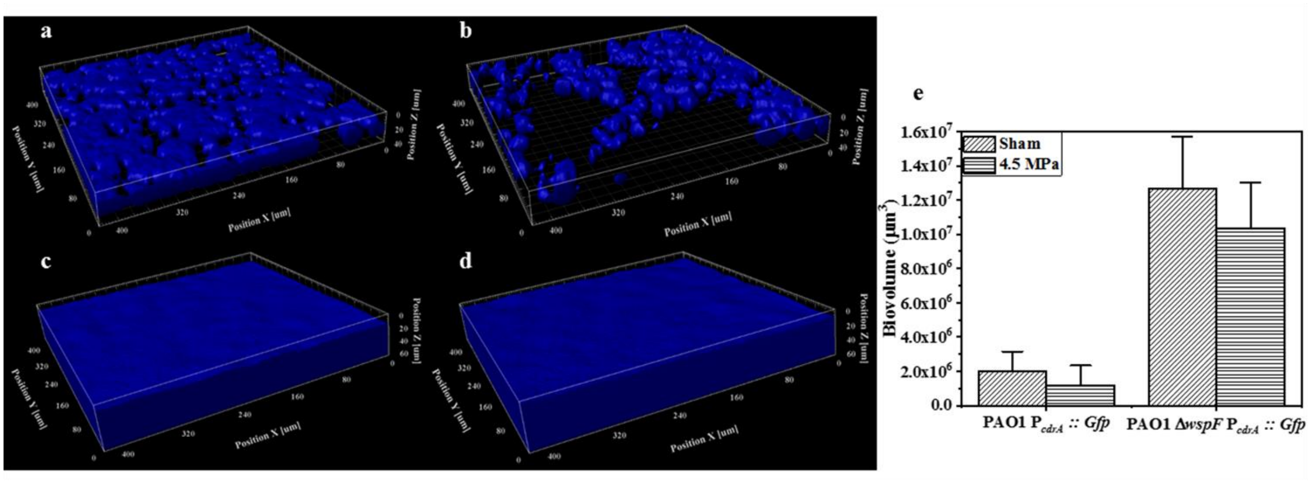

Supplementary Figure 3. 3D reconstructions of biofilms formed by **a:** wild type strain – untreated **b:** wild type strain – HIFU-treated **c:** ΔwspF strain – untreated and **d:** ΔwspF strain – HIFU-treated. **e:** Quantitative analysis – biovolume of the untreated and treated biofilms (WT untreated : N=13, Mean ± SD; WT treated : N=7, Mean ± SD; ΔwspF untreated and treated: N=6, Mean ± SD)

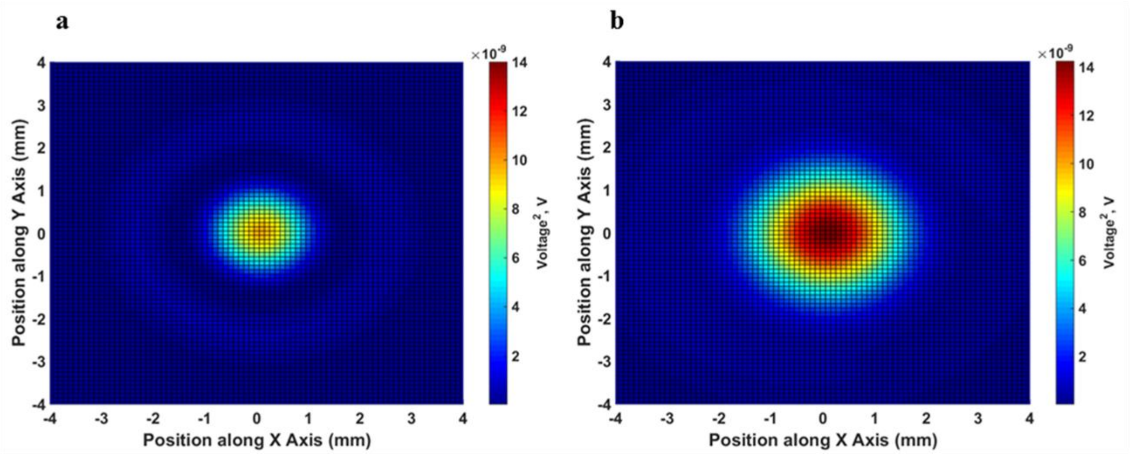

Supplementary Figure 4. Pressure distribution of 0.5 MHz transducer in **a:** free-field and **b:** in presence of a coupling cone sealed with Mylar

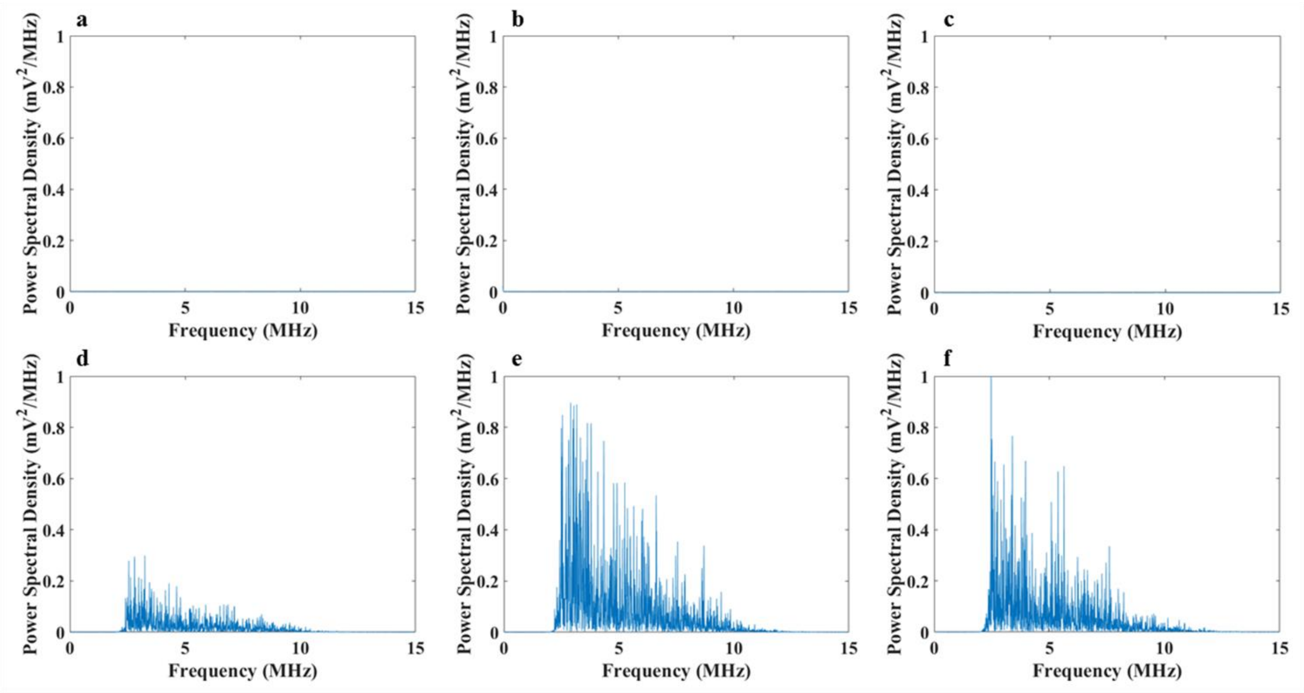

Supplementary Figure 5. Inertial cavitation response: broadband signal at **a:** 0.5 MPa **b:** 1.5 MPa **c:** 2.5 MPa **d:** 3.5 MPa **e:** 4.5 MPa **f:** 5.5 MPa.

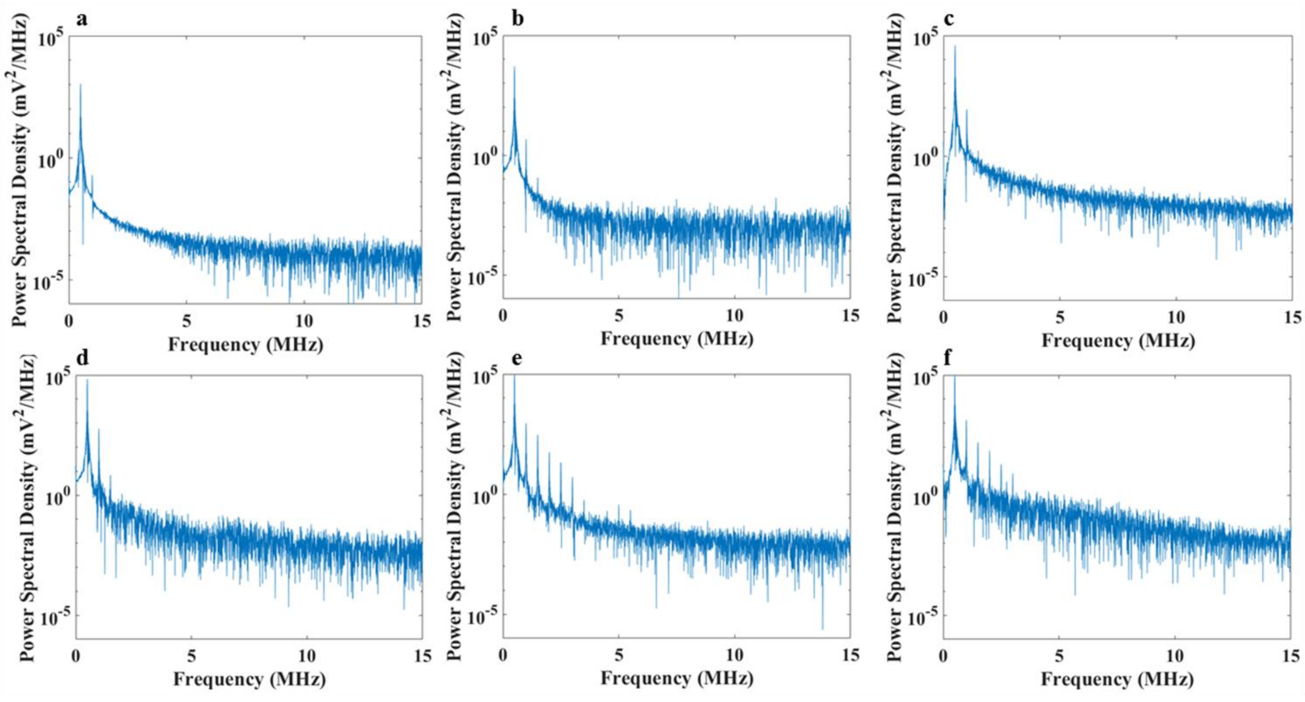

Supplementary Figure 6. Stable cavitation response: sub-harmonic signal at **a:** 0.5 MPa **b:** 1.5 MPa **c:** 2.5 MPa **d:** 3.5 MPa **e:** 4.5 MPa **f:** 5.5 MPa.

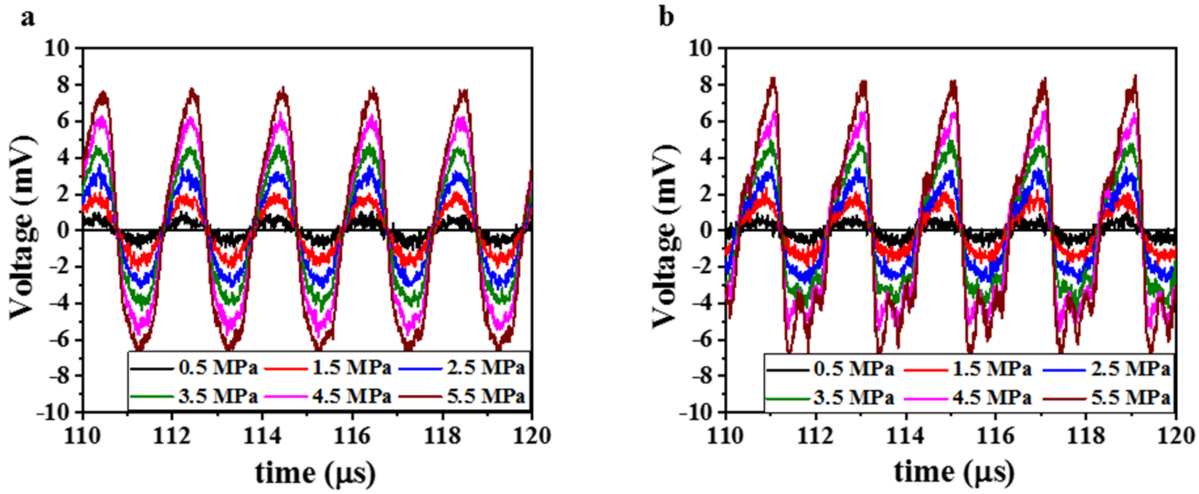

Supplementary Figure 7. Acoustic wave propagation at various acoustic pressures in **a:** free-field and **b:** in presence of a coupling cone sealed with Mylar

2 Supplementary data: MATLAB codes for image processing and analysis

Code snippets for image processing and quantification of confocal images are provided below:

2.1 Volumetric parameters determination

Example of Channel 1 calculation:

```
%Define 3D matrix to store processed image stack
%Ch1_Data is the 3D confocal image matrix
Thresh_Filt_Stack = zeros (size(Ch1_Data), 'uint8');

sliceLength = size(Ch1_Data, 3); %z-direction
for slice = 1:sliceLength
Layer = Ch1_Data(:,:,slice);

%Threshold graylevel images using inbuilt Otsu’s thresholding
%function from MATLAB Image processing toolbox
[levelOT] = graythresh(Layer);
Ch1_Data_thresh = im2bw(Layer,levelOT);
Ch1_Data_thresh = uint8(Ch1_Data_thresh);

%Filter binary images using inbuilt 2D median filtering
%function from MATLAB Image processing toolbox
Ch1_Data_filt = medfilt2(Ch1_Data_thresh,[5 5]);

%Assign the processed image to 3D matrix
Thresh_Filt_Stack(:,:,slice) = Ch1_Data_filt;

end

%Calculate biovolume of processed 3D stack
%Function defined by Lewandowski, Z., and Beyenal, H.
%Fundamentals of Biofilm Research, 2007.
voxVolScaling = info.voxSizeX*info.voxSizeY*10^12 * info.voxSizeZ*10^6;
Biovolume = sum(Thresh_Filt_Stack(:))*voxVolScaling;

%Calculate porosity = total void pixels/total number of pixels
%Function defined by Lewandowski, Z., and Beyenal, H.
%Fundamentals of Biofilm Research, 2007.
Total_pixels = prod(Thresh_Filt_Stack);
Porosity = 1 - (Biovolume/ Total_pixels);

%Calculate run lengths in X,Y and Z directions
%Function defined by Lewandowski, Z., and Beyenal, H.
%Fundamentals of Biofilm Research, 2007.
[AXRL,AYRL,AZRL] = runLengths3D(Thresh_Filt_Stack);
AXRL = AXRL*info.voxSizeX*10^6;
AYRL = AYRL*info.voxSizeY*10^6;
AZRL = AZRL*info.voxSizeZ*10^6;
```

2.2 Gfp/Cfp ratio determination

For 3D data quantification:

```
%Define 3D matrices for storage
BIO_Filt_Stack = zeros(size(Ch1_Data)); %For binary biovolume data
Gfp_Stack = zeros(size(Ch2_Data)); %For graylevel Gfp data
Cfp_Stack = zeros(size(Ch1_Data)); %For graylevel Cfp data

sliceLength = size(Ch1_Data, 3);

for slice = 1:sliceLength
   Layer = Ch1_Data(:,:,slice);
   Layer1 = Ch2_Data(:,:,slice);

%Convert to double to allow calculations between two images
   CFPLayer = double(Layer);
   GFPLayer = double(Layer1);

%Create binary mask by thresholding graylevel images
%and filtering binary images
[levelOT] = graythresh(Layer);
CFP_Data_thresh = imbinarize(Layer,levelOT);
CFP_Data_thresh = double(CFP_Data_thresh);
CFP_Data_filt = medfilt2(CFP_Data_thresh,[5 5]);
CFP_Filt_Stack(:,:,slice) = CFP_Data_filt;

%Apply mask to Gfp data
GFP_mask = GFPLayer.*CFP_Data_filt;
GFP_Stack(:,:,slice) = GFP_mask;

%Apply mask to Cfp data
BIO_mask = CFPLayer.*CFP_Data_filt;
CFP_Stack(:,:,slice) = BIO_mask;
end

%Quantify the ratio
GFP_Ints = sum(GFP_Stack(:));
CFP_Ints = sum(CFP_Stack(:));
GFPCFPRatio = GFP_Ints/CFP_Ints;
```

For qualitative ratiometric images:

```
%%As described by Nair et al. (2017); doi: 10.1074/jbc.M116.746743
%Create a Max Z-Projection of the Z-stacked image data
GFP_MaxZ = max(Ch2_Data,[],3);
GFP_MaxZ = double(GFP_MaxZ);

CFP_Max = max(Ch1_Data,[],3);
CFP_MaxZ = double(CFP_Max);

%Obtain the ratiometric image
GFPCFP_RatioImage = GFP_MaxZ./CFP_MaxZ;

%%Auto Threshold the Biomass data with Otsu
```

```
[level1] = graythresh(CFP_Max);
BIO_OTbw = im2bw(CFP_Max,level1);
BIO_OTbw = uint8(BIO_OTbw);

%%Filter the image with filter size = 5
BIO_fl = medfilt2(BIO_OTbw,[5 5]);
BIO_fl = double(BIO_fl);

% Apply binary mask to ratiometric image
GFPBIO_mask = GFPBIO_RatioImage.*BIO_fl;

%Get the final ratiometric image
 c = jet(16);
 imagesc(GFPBIO_mask);
 colormap(c);
```
